# Early refractive stability and predictive accuracy of a novel hydrophobic C-loop monofocal intraocular lens in eyes with normal axial length

**DOI:** 10.1097/MD.0000000000050119

**Published:** 2026-08-07

**Authors:** Dong Gyu Na, Hyeck-Soo Son, Hungwon Tchah, Kyungmin Koh

**Affiliations:** a Department of Ophthalmology, Kim’s Eye Hospital, Konyang University College of Medicine, Seoul, Republic of Korea; b Department of Ophthalmology, University of Heidelberg, David J. Apple International Laboratory for Ocular Pathology and International Vision Correction Research Centre, Heidelberg, Germany.

**Keywords:** C-loop haptic, cataract surgery, early postoperative refraction, hydrophobic IOL, monofocal intraocular lens, refractive stabilization

## Abstract

This study aimed to evaluate the early postoperative refractive stabilization pattern and time course associated with a novel hydrophobic C-loop monofocal intraocular lens (IOL) following phacoemulsification cataract surgery. This retrospective study evaluated patients who underwent uneventful phacoemulsification and implantation of a single-piece hydrophobic C-loop monofocal IOL. Manifest refraction spherical equivalent (MRSE) and refractive error (RE) were assessed on postoperative day 1, at 1 week, and at 1 month. Primary outcomes included longitudinal changes in MRSE and RE to evaluate refractive stabilization over time. Secondary outcomes included prediction error and the proportion of eyes achieving target refraction accuracy. The study included 43 eyes of 43 patients with a mean age of 69.9 ± 6.3 years, including 24 females (55.8%). A statistically significant myopic shift was observed during the first postoperative month; the mean MRSE progressively shifted from 0.08 ± 0.50 D on day 1 to −0.09 ± 0.40 D at 1 week, and stabilized at −0.19 ± 0.42 D at 1 month (*P* < .001). Correspondingly, the mean RE shifted from 0.25 ± 0.52 D on day 1 to 0.08 ± 0.41 D at 1 week, and −0.02 ± 0.43 D at 1 month (*P* < .001). Regarding refractive accuracy, the proportion of eyes within ± 0.50 D of the target refraction significantly improved from 51.2% on day 1 to 76.7% at 1 week and 74.4% at 1 month (*P* < .001). The mean absolute error also decreased significantly from 0.49 ± 0.30 D on day 1 to 0.36 ± 0.24 D at 1 month (*P* < .001). No intraoperative or postoperative complications were observed. This novel hydrophobic C-loop monofocal IOL exhibits a predictable and progressive early refractive pattern characterized by an initial myopic shift within the first postoperative week, followed by stabilization up to 1 month. These findings support the clinical feasibility of functional refraction and spectacle prescription as early as 1 week following uncomplicated cataract surgery.

## 1. Introduction

The increased life expectancy and dramatically improved cataract surgery techniques have led to an increase in number of patients undergoing cataract surgery.^[1]^ Modern phacoemulsification with monofocal intraocular lenses (IOLs) provides highly predictable postoperative visual and refractive outcomes, yet early postoperative refraction is not immediately stable.^[2]^ Postoperative refraction is a primary driver of patient satisfaction and is largely determined by the orientation of the IOL within the capsular bag.^[3]^ This alignment is significantly affected by various factors, including the dimensions of the capsular bag, the specific IOL design, the surgical rhexis size and shape, and the mechanical interaction between the lens and the capsule after implantation.^[4]^ Multiple studies have demonstrated that the largest refractive shift typically occurs between postoperative day 1 and week 1, followed by a smaller and slower change up to 1 month, after which refractive stability is usually achieved.^[5,6]^

Determining the refractive stabilization period for newly introduced intraocular lenses is of great clinical importance.^[7]^ This timing is a key factor that directly influences spectacle prescription, second-eye surgery planning, and overall refractive predictability. While traditional protocols suggest waiting 4 to 6 weeks postoperatively, recent evidence indicates that functional stability can be achieved as early as 1 week in uncomplicated monofocal cases.^[8–10]^

The newly released monofocal CT LUCIA 621P (621P; Carl Zeiss Meditec AG, Jena, Germany) IOL has been reported in several studies to provide superior intermediate visual acuity (VA) compared with conventional monofocal IOLs.^[2,11]^

This study aimed to evaluate the early refractive stability and predictive accuracy of the 621P IOL to determine the optimal timing for reliable postoperative outcomes.

## 2. Materials and methods

### 2.1. Participants

This retrospective, single center, non-randomized study abided by the Declaration of Helsinki, and was given approval by the Institutional Review Board of the Kim’s Eye Hospital, Seoul, Republic of Korea (approval number: 2026-03-004). The IRB waived written informed consent as the data from the retrospective study was reviewed anonymously. Cataract patients who underwent implantation of the 621P IOL between November 2024 and April 2025 were included in this study. Participants younger than 50 years of age, with corneal astigmatism of ≥1.00 D, or with an axial length (AL) of <22.0 mm or > 25.0 mm were excluded.^[10]^ This range was specifically selected to exclude eyes with extreme axial dimensions, thereby minimizing potential confounding errors in effective lens position (ELP) prediction formulas and ensuring a highly homogeneous cohort to precisely evaluate early refractive stability. Other exclusion criteria encompassed significant ophthalmic conditions that might interfere with capsular bag stability or visual function, including pseudoexfoliation syndrome, prior ocular trauma, and dense corneal scarring. Additionally, patients were excluded if they presented with risk factors for intraoperative floppy iris syndrome, any systemic or ocular pathologies beyond cataracts that could impair vision, or a history of previous intraocular, corneal, or refractive procedures. Any cases involving intraoperative or postoperative complications that compromised visual results were also omitted from the study. To minimize the influence of individual patient factors only the first operated eye was included in cases of bilateral surgery. The VA was measured using the Early Treatment Diabetic Retinopathy Study (ETDRS) chart and recorded in logarithm of the Minimum Angle of Resolution (logMAR) units. Both uncorrected distance visual acuity (UDVA) and corrected distance visual acuity (CDVA) were assessed at a testing distance of 6 meters. VA testing was confined to a maximum measurable value of 0.0 logMAR.

### 2.2. Intraocular lens

Available as a fully preloaded system, the 621P is a single-piece aspheric monofocal IOL comprising a 6.0-mm optic with a heparin-coated surface and a continuous 360-degree square posterior edge.^[3,12]^ Measuring 13.0 mm in overall length, the device utilizes step-vaulted C-loop haptics to maximize capsular bag stability.^[2]^ Optically, the lens applies central negative spherical aberration (SA) to counteract the positive SA of the cornea, while the peripheral optic incorporates positive SA to mitigate the visual consequences of lens displacement.^[13]^ Provided in 0.5 D steps from 0.0 D to 34.0 D, this configuration is intentionally designed to maintain robust optical performance even in the presence of mild postoperative decentration.^[2,14]^

### 2.3. Pre- and postoperative assessment

All patients received a comprehensive preoperative ophthalmic evaluation, which included a slit-lamp examination (SLE), manifest refraction (MR), and assessment of UDVA and CDVA. Biometric parameters, including AL, anterior chamber depth (ACD), white-to-white distance, lens thickness, keratometry, and corneal astigmatism, were measured using the IOL Master 700 (Carl Zeiss Meditec AG, Germany). This swept-source optical coherence tomography device simultaneously captures axial dimensions and evaluates anterior keratometry using a telecentric 3-ring method to generate simulated *K* values within a 2.5-mm optical zone.^[15]^ The Barrett Universal II formula was utilized for all IOL power calculations, with the refractive target set to emmetropia.

Postoperative follow-up examinations were scheduled at 1 day, 1 week, and 1 month after surgery. Each visit involved a thorough ophthalmic assessment, including SLE, MR, and monocular measurements of UDVA and CDVA to determine the postoperative refractive state. In this study, early refractive stability was defined as the postoperative time point at which longitudinal changes in MRSE became clinically negligible (≤0.10 D), accompanied by a plateau in the proportion of eyes achieving within ± 0.50 D of the target refraction.

Refractive error (RE) was calculated as the difference between the postoperative manifest refraction spherical equivalent (MRSE) and the intended target SE.^[16]^ Mean absolute error (MAE) was then determined by averaging these absolute RE values.^[17]^ Furthermore, any intraoperative and postoperative complications were closely monitored and recorded throughout the observation period.

### 2.4. Surgical procedures

Every cataract procedure was performed by 1 veteran surgeon (KK) employing the Whitestar Signature^®^ phacoemulsification system (Johnson & Johnson Vision, Santa Ana). A 2.8-mm clear corneal incision was positioned on the steep axis, after which a continuous curvilinear capsulorhexis of 5.2 mm was executed. Following lens extraction and IOL placement, the ophthalmic viscosurgical device (Provisc^®^; Alcon Laboratories, Fort Worth) was thoroughly aspirated. As the final step of the procedure, stromal hydration was applied to seal the wounds. Following the surgery, a regimen of topical medications was prescribed. Patients were directed to administer 0.3% gatifloxacin (Gatiflo; Samil Co., Ltd., Seoul, South Korea) and 0.1% fluorometholone (Fulleylone; Binex Co., Busan, South Korea) 4 times a day until 1 month postoperatively.

### 2.5. Statistical analysis

Statistical analysis of the data was conducted using IBM SPSS Statistics for Windows, version 22.0 (IBM Corporation, Armonk). Descriptive statistics were presented as means and standard deviations with 95% confidence intervals (CIs) for continuous variables, and as frequencies and percentages for categorical variables. The Wilcoxon signed-rank test was utilized to compare continuous variables across the 3 postoperative time points, and Cohen *d* was calculated to evaluate the standardized effect size of longitudinal changes. For categorical variables, Cochran *Q* test was employed to assess overall differences across the time points. Subsequent post hoc pairwise comparisons were conducted using the McNemar test. To adjust for multiple comparisons across the 3 time points, a Bonferroni correction was applied, setting the threshold for statistical significance at *P* < .0167. Finally, a priori power analysis was performed based on the observed longitudinal changes in MRSE and RE to verify whether the sample size of 43 eyes provided sufficient statistical weight.

## 3. Results

### 3.1. Patient demographics and baseline characteristics

A total of 43 eyes from 43 patients were included in this study. The mean age of the patients was 69.9 ± 6.3 years, ranging from 55 to 84 years. The study cohort comprised 24 females (55.8%), and surgery was performed on 26 left eyes (60.5%). Preoperative ocular biometry revealed a mean AL of 23.52 ± 0.76 mm and a mean ACD of 3.17 ± 0.40 mm. The mean target spherical equivalent was −0.18 ± 0.22 D. Detailed baseline demographic and ocular characteristics are summarized in Table 1. A priori power analysis based on the longitudinal changes in refractive outcomes confirmed that the sample size of 43 eyes provided a statistical power exceeding 95% at an alpha level of 0.05. All surgical procedures were completed without intraoperative complications, and no significant adverse events were observed during the 1-month follow-up period.

**Table 1:** Baseline patient demographics and preoperative biometry.

Variables	
Patients/eyes, n	43/43
Age (yr)	69.9 ± 6.3 (55–84)
Female, n	24 (55.8%)
Left eye, n	26 (60.5%).
Anterior chamber depth (mm)	3.17 ± 0.40 (2.38–3.93)
Axial length (mm)	23.52 ± 0.76 (22.24–25.44)
White to white (mm)	11.90 ± 0.41 (11.10–12.90)
Lens thickness (mm)	4.56 ± 0.38 (3.63–5.45)
Flat keratometry (D)	43.85 ± 1.47 (41.30–46.46)
Steep keratometry (D)	44.40 ± 1.43 (41.90–47.32)
Astigmatism (D)	0.55 ± 0.29 (0.12–0.94)
Target SE (D)	−0.18 ± 0.22 (−0.31 to 0.18)

Values are presented as mean ± SD.

D = diopter, n = number, SD = standard deviation, SE = spherical equivalent.

### 3.2. Visual acuity outcomes

Clinical outcomes, including 95% confidence intervals (CIs) and effect sizes across postoperative time points, are comprehensively summarized in Table 2. UDVA showed continuous and statistically significant improvement over the postoperative period, improving from 0.06 ± 0.04 logMAR [95% CI: 0.05, 0.07] at 1 day to 0.05 ± 0.03 logMAR [95% CI: 0.04, 0.06] at 1 week, and reaching 0.03 ± 0.03 logMAR [95% CI: 0.02, 0.04] at 1 month (*P* < .001). CDVA also demonstrated an overall significant change, measuring 0.01 ± 0.02 logMAR [95% CI: 0.00, 0.02] at 1 day and reaching 0.00 ± 0.01 logMAR [95% CI: 0.00, 0.00] by 1 month (*P* < .025). Although the overall comparison of CDVA showed a statistically significant change, post hoc pairwise comparisons using Bonferroni correction revealed no significant differences between any specific consecutive time points.

**Table 2:** Comparison of clinical outcomes across postoperative time points.

Parameter	1 day (D)	1 week (W)	1 month (M)	*P*-value	Effect size (Cohen *d*)
UDVA (logMAR)	0.06 ± 0.04 a [95% CI: 0.05, 0.07]	0.05 ± 0.03 b [95% CI: 0.04, 0.06]	0.03 ± 0.03 c [95% CI: 0.02, 0.04]	**<.001**	0.28 (1D to 1W) 0.67 (1W to 1M)
CDVA (logMAR)	0.01 ± 0.02 a [95% CI: 0.00, 0.02]	0.01 ± 0.01 a [95% CI: 0.01, 0.01]	0.00 ± 0.01 a [95% CI: 0.00, 0.00]	**<.025**	0.00 (1D to 1W) 1.00 (1W to 1M)
MRSE (D)	0.08 ± 0.50 a [95% CI: −0.07, 0.23]	−0.09 ± 0.40 b [95% CI: −0.21, 0.03]	−0.19 ± 0.42 c [95% CI: −0.32, −0.06]	**<.001**	0.38 (1D to 1W) 0.24 (1W to 1M)
RE (D)	0.25 ± 0.52 a [95% CI: 0.09, 0.41]	0.08 ± 0.41 b [95% CI: −0.05, 0.21]	−0.02 ± 0.43 c [95% CI: −0.15, 0.11]	**<.001**	0.36 (1D to 1W) 0.24 (1W to 1M)
MAE (D)	0.49 ± 0.30 a [95% CI: 0.40, 0.58]	0.35 ± 0.23 b [95% CI: 0.28, 0.42]	0.36 ± 0.24 b [95% CI: 0.29, 0.43]	**<.001**	0.52 (1D to 1W) 0.04 (1W to 1M)

CDVA = corrected distance visual acuity, CI = confidence interval, D = diopter, logMAR = logarithm of the minimum angle of resolution, MAE = mean absolute error, MRSE = manifest refraction spherical equivalent, RE = refractive error, UDVA = uncorrected distance visual acuity.

Values are presented as mean ± SD with 95% CIs in brackets. *P*-values in the rightmost column for comparison between 1 day and 1 month were calculated using the Wilcoxon signed-rank test for continuous variables. Different superscript letters (a, b, c) in the same row indicate statistically significant differences between time points (*P* < .0167, Wilcoxon signed-rank test with Bonferroni correction). Cohen *d* was calculated to evaluate the standardized effect size of longitudinal changes between consecutive follow-up intervals.

Bold values indicate statistically significant results (*P* < .05).

### 3.3. Refractive outcomes and myopic shift

**Figure 1.**
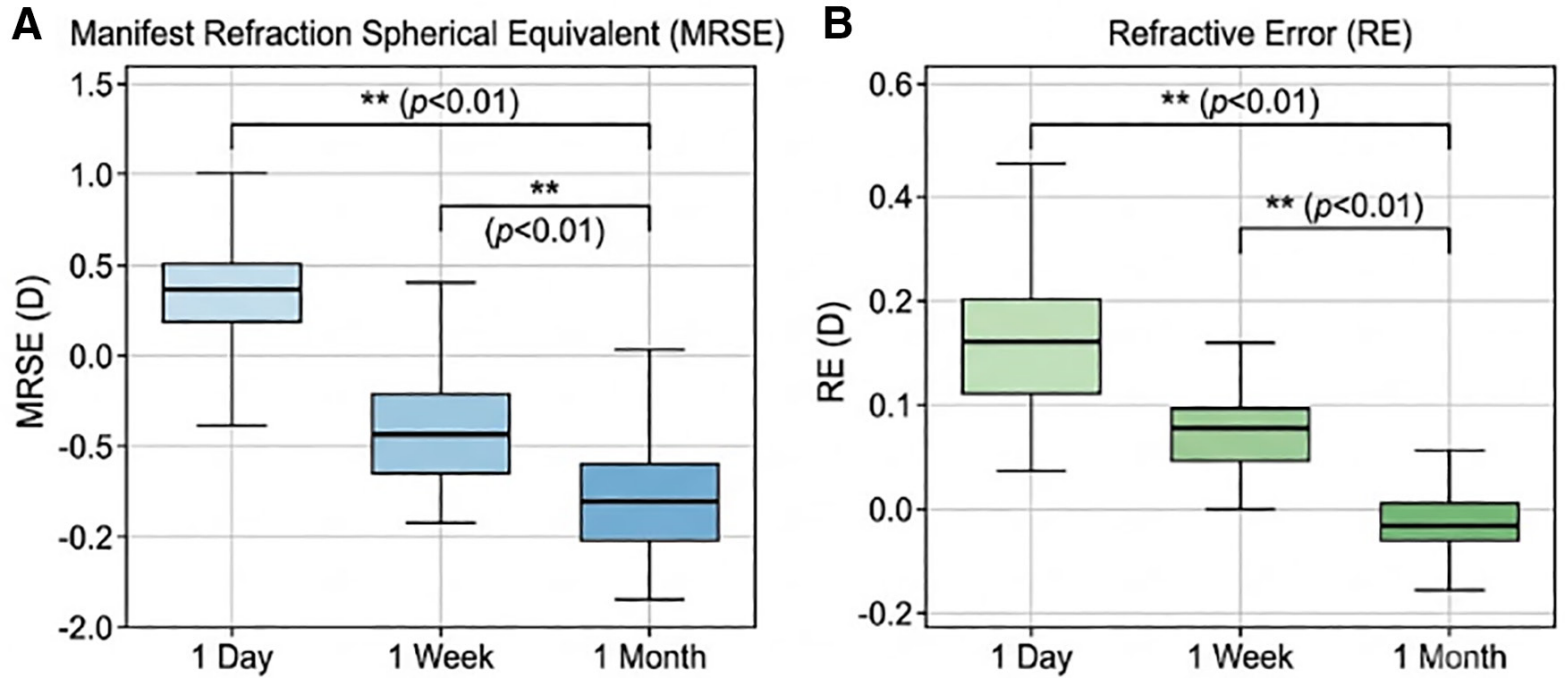
Longitudinal changes in postoperative refractive outcomes at 1 day, 1 week, and 1 month. (A) Box-and-whisker plot illustrating the distribution of manifest refraction spherical equivalent (MRSE). (B) Box-and-whisker plot of the refractive error (RE). Both plots demonstrate a progressive and statistically significant narrowing of variability and stabilization over the 1-month follow-up period. ** indicates a statistically significant difference between the time points (*P* < .01) based on pairwise comparisons with Bonferroni correction. MRSE = manifest refraction spherical equivalent, RE = refractive error.

Analysis of the refractive outcomes revealed a progressive and statistically significant myopic shift throughout the early follow-up period, followed by functional stabilization. As detailed in Table 2, the mean MRSE shifted from 0.08 ± 0.50 D [95% CI: −0.07, 0.23] on day 1 to −0.09 ± 0.40 D [95% CI: −0.21, 0.03] at 1 week. While a minor statistical difference was observed between 1 week and 1 month (−0.19 ± 0.42 D [95% CI: −0.32, −0.06]; *P* < .001), this subsequent interval change (−0.10 D) was clinically negligible (Cohen *d* = 0.24) and fell well within standard measurement tolerance (Fig. 1A). Similarly, the mean RE demonstrated a corresponding shift from 0.25 ± 0.52 D [95% CI: 0.09, 0.41] on day 1 to 0.08 ± 0.41 D [95% CI: −0.05, 0.21] at 1 week and stabilized at −0.02 ± 0.43 D [95% CI: −0.15, 0.11] at 1 month (*P* < .001; Fig. 1B).

### 3.4. Refractive accuracy

Refractive accuracy demonstrated a significant improvement after the first postoperative day and plateaued thereafter, confirming early stability. The mean absolute error significantly decreased from 0.49 ± 0.30 D [95% CI: 0.40, 0.58] at day 1 to 0.35 ± 0.23 D [95% CI: 0.28, 0.42] at week 1, and remained virtually unchanged at 0.36 ± 0.24 D [95% CI: 0.29, 0.43] at month 1 with a minimal effect size (Cohen *d* = 0.04; Table 2, *P* < .001). As shown in Table 3, the percentage of eyes achieving an MRSE within 0.50 D of the target was 51.2% [95% CI: 36.8, 65.4] at day 1, which significantly increased to 76.7% [95% CI: 62.3, 86.8] at week 1 and maintained a stable plateau at 74.4% [95% CI: 59.8, 85.1] at month 1 (*P* < .001). Furthermore, the proportion of eyes within 0.75 D improved significantly from 76.7% [95% CI: 62.3, 86.8] at day 1 to 97.7% [95% CI: 87.9, 99.6] at week 1, maintaining 95.3% [95% CI: 84.5, 98.7] at month 1 (*P* < .001). By 1 month postoperatively, all 43 eyes (100.0%) achieved a spherical equivalent within 1.00 D of the intended target.

**Table 3:** Postoperative refractive accuracy.

Parameter	1 day	1 week	1 month	*P*-value
MRSE within 0.5 D (n [%])	22 (51.2%) a [95% CI: 36.8, 65.4]	33 (76.7%) b [95% CI: 62.3, 86.8]	32 (74.4%) b [95% CI: 59.8, 85.1]	**<.001**
MRSE within 0.75 D (n [%])	33 (76.7%) a [95% CI: 62.3, 86.8]	42 (97.7%) b [95% CI: 87.9, 99.6]	41 (95.3%) b [95% CI: 84.5, 98.7]	**<.001**
MRSE within 1.0 D (n [%])	42 (97.7%) a [95% CI: 87.9, 99.6]	42 (97.7%) a [95% CI: 87.9, 99.6]	43 (100.0%) a [95% CI: 91.8, 100.0]	.368

Values are presented as number (%). *P*-values in the rightmost column were calculated using Cochran *Q* test. Different superscript letters (a, b, c) in the same row indicate statistically significant differences between time points (*P* < .0167, McNemar test with Bonferroni correction). Values in brackets represent 95% CIs for the proportions.

Bold values indicate statistically significant results (*P* < .05).

CI = confidence interval, D = diopter, MRSE = manifest refraction spherical equivalent.

## 4. Discussion

This study assessed the clinical performance of the 621P IOL in a real-world clinic setting. The results demonstrated favorable visual and refractive outcomes, with 100.0% of eyes showing UDVA within 1 line of CDVA, and 39.5% achieving a UDVA of 0.0 logMAR at 1 month postoperatively. In an in vitro analysis using cadaveric eyes, the 621PY IOL demonstrated an extended contact arc with the capsule and an absence of capsular striae compared to other lens models.^[18]^ The structural characteristics of the 621PY IOL, including its sharp-edged haptics and the extensive contact area at the optic-haptic junction, suggest both excellent intracapsular stability and a potential for lower rates of posterior capsular opacification.^[19]^

In this cohort of 43 eyes, there was a tendency for a myopic shift during the early postoperative period. The mean postoperative MRSE changed from 0.08 ± 0.50 D on postoperative day 1 to −0.09 ± 0.40 D at week 1, and −0.19 ± 0.42 D at month 1. The largest shift in SE (−0.17 D) occurred between day 1 and week 1 (*P* = .002), followed by a smaller additional change (−0.10 D) between week 1 and month 1 (*P* = .007). This trend is consistent with prior studies indicating that the most significant refractive shifts occur within the first postoperative week, with only marginal changes observed subsequently.^[6,8,9]^ Sugar et al documented a shift from +0.31 D on day 1 to −0.33 D at week 1 and −0.51 D at 1 month, while Ostri et al confirmed that automated refraction typically reaches stability by day 7 in most cases.^[8,20]^

In another study, the mean spherical error was 0.37 ± 0.75 D at 2 weeks and 0.33 ± 0.73 D at 6 weeks, with a mean difference of 0.04 ± 0.30 D. Similarly, the mean cylindrical error was −0.40 ± 0.68 D at 2 weeks and −0.43 ± 0.66 D at 6 weeks, showing a minimal difference of 0.03 ± 0.40 D.^[7]^ These findings confirm that both spherical and cylindrical refractions achieve stability between the second and sixth weeks postoperatively. Furthermore, a separate study evaluating the refractive stability of 116 myopic eyes implanted with a monofocal IOL showed that subjective refraction reached stabilization within 1 week postoperatively, showing no significant differences in mean spherical (*P* = .33), cylindrical (*P* = .65), or spherical equivalent (*P* = .45) refractions between the 1-week and 1-month visits.^[21]^

Two major mechanisms have been proposed to explain this early postoperative refractive drift. The first is corneal stromal edema, which occurs secondary to ultrasound energy and transient endothelial dysfunction during surgery. This edema temporarily alters both anterior and posterior corneal curvature, resulting in either hyperopic or myopic shifts that usually resolve within 1 to 2 weeks. The second is ELP change. Early postoperative variations in ACD and IOL axial positioning have been shown to influence refractive outcomes. Prospective anterior segment imaging studies have demonstrated that most IOL positional changes occur during the first postoperative week, with minimal movement thereafter.^[10,22]^

The initial hyperopic-to-myopic drift observed in our study, shifting from a mean SE of 0.08 D on day 1 to −0.09 D at week 1, is consistent with the resolution of corneal edema during the first postoperative week.^[23,24]^ The wide distribution of SE on day 1 (−1.00 D to +0.88 D) reflects the variable impact of early corneal swelling. By week 1, this variability decreased, consistent with previous observations showing corneal clarity and curvature normalization within 1 to 2 weeks.^[23]^ Similar findings have been reported in previous studies. In a cohort of 163 eyes, RE stabilization was achieved by postoperative day 28 in 98.8% of cases.^[10]^

The small residual myopic shift observed from week 1 to month 1 is hypothesized to be related to the stabilization of the effective lens position. Previous studies using anterior segment imaging have documented measurable axial movements of the IOL and reductions in ACD during the early postoperative period, with the majority of changes occurring during the first week and stabilizing thereafter.^[23,25,26]^ Our findings align closely with these reports.

Previous studies evaluating various monofocal IOLs have reported predictable clinical outcomes. For instance, the LI61AO Sofport^®^ (Bausch and Lomb, Inc., Rochester) IOL demonstrated a mean SE of 0.20 ± 0.63 D at 1 month, with 38% and 64% of eyes within 0.25 D and 0.50 D of the target emmetropia, respectively.^[27]^ Similarly, the Clareon^®^ (Alcon, Inc., Fort Worth) IOL showed a 1-month mean UDVA of 0.05 ± 0.12 logMAR and an SE of −0.19 ± 0.38 D.^[28]^ Furthermore, the Artis PL E (Cristalens Industrie, Lannion, France) IOL achieved an SE of -0.21 ± 0.34 D at 3 months postoperatively. Regarding refractive predictability, the mean RE was 0.04 ± 0.33 D, and the mean absolute error (MAE) was 0.26 ± 0.21 D.^[29]^

Regarding the 621P IOL specifically, existing clinical data have established its strong baseline stability and predictable postoperative outcomes. In a study evaluating 74 eyes of 37 patients, the mean postoperative CDVA reached − 0.08 ± 0.08 logMAR after femtosecond laser-assisted cataract surgery and 0.08 ± 0.07 logMAR after conventional cataract surgery, with no statistically significant differences between techniques. The MAE (0.48 ± 0.38 D) and RE (0.42 ± 0.44 D) also indicated good refractive accuracy and stability (*P* = .559 and *P* = .786).^[3]^ Additionally, Schallhorn et al evaluated the 621P IOL over a mean follow-up of 1.7 months, reporting a mean UDVA of 0.09 ± 0.16 logMAR. In their cohort, 74.9% of eyes achieved a UDVA of 20/25 or better, and 91.1% reached a CDVA of 20/25 or better. In terms of refractive predictability, 84.8% and 98.4% of eyes were within 0.50 D and 1.00 D of the emmetropic target, respectively.^[30]^ This finding is consistent with our results, further supporting the stable refractive performance of the 621P IOL in routine clinical settings.

Compared to these established models and prior 621P reports, our results confirm that the 621P IOL provides highly competitive refractive accuracy in a routine clinical setting. Our cohort achieved a lower mean absolute error of 0.36 ± 0.24 D and excellent predictability, with 74.4% of eyes falling within 0.50 D of the intended target and 100% achieving a spherical equivalent within 1.00 D at 1 month postoperatively.

These results hold direct clinical implications. The demonstrated refractive stability of the 621P IOL at 1 week supports performing functional refraction early in the postoperative period for most uncomplicated cases.^[7]^ This facilitates timely spectacle prescription and flexible planning for sequential second-eye surgery. However, in cases involving compromised endothelium, excessive phacoemulsification energy, or complex surgical procedures, delaying final refraction until 3 to 4 weeks remains prudent.^[22,31]^

Several limitations must be acknowledged. First, the retrospective design and relatively small sample size may introduce selection bias. Second, the absence of a parallel control or comparator group remains a major limitation, which warrants caution when interpreting the comparative superiority or clinical performance of these outcomes relative to other platforms. Third, visual acuity testing was capped at 0.0 logMAR; this ceiling effect may have underestimated the true visual potential of the 621P IOL by failing to differentiate eyes capable of achieving better than 20/20 vision. Finally, we did not directly measure longitudinal changes in central corneal thickness, ACD, or the precise anatomical position of the IOL using objective anterior segment imaging. These early refractive changes likely result from the resolution of subclinical corneal edema and effective lens position settling. Therefore, future prospective trials utilizing advanced anterior segment imaging are necessary to precisely quantify their respective contributions.

In conclusion, the early postoperative refractive changes following 621P IOL implantation exhibit a predictable trajectory, characterized by a progressive myopic shift between postoperative day 1 and week 1, with minimal subsequent drift up to 1 month. This pattern aligns with the physiological resolution of corneal edema and the stabilization of the effective lens position. Although the absence of a parallel comparator group limits direct comparative conclusions, our longitudinal findings consistently support the clinical feasibility of functional refraction and spectacle prescription as early as 1 week following uncomplicated surgery.

## Author contributions

**Conceptualization:** Hungwon Tchah, Kyungmin Koh.

**Data curation:** Dong Gyu Na.

**Formal analysis:** Hyeck-Soo Son.

**Investigation:** Dong Gyu Na, Hyeck-Soo Son.

**Methodology:** Dong Gyu Na, Hungwon Tchah.

**Project administration:** Kyungmin Koh.

**Resources:** Hungwon Tchah, Kyungmin Koh.

**Software:** Dong Gyu Na.

**Validation:** Dong Gyu Na, Hyeck-Soo Son.

**Visualization:** Dong Gyu Na.

**Writing – original draft:** Dong Gyu Na, Kyungmin Koh.

**Writing – review & editing:** Hyeck-Soo Son, Hungwon Tchah, Kyungmin Koh.
